# Pleomorphic lobular carcinoma of the breast with osteoclast-like giant cells: a case report and review of the literature

**DOI:** 10.1186/s13000-018-0744-6

**Published:** 2018-08-28

**Authors:** Lourdes Peña-Jaimes, Irene González-García, María Eugenia Reguero-Callejas, Ignacio Pinilla-Pagnon, Belén Pérez-Mies, Víctor Albarrán-Artahona, Noelia Martínez-Jañez, Juan Manuel Rosa-Rosa, José Palacios

**Affiliations:** 1 Department of Pathology, Hospital Ramón y Cajal, Madrid, Spain; 2 Department of Oncology, Hospital Ramón y Cajal, Madrid, Spain; 3 CIBER-ONC, Insituto de Salud Carlos III, Madrid, Spain; 4 Instituto Ramón y Cajal de Investigación Sanitaria, Madrid, Spain; 5 Universidad de Alcalá de Henares, Madrid, Spain

**Keywords:** Pleomorphic carcinoma, Osteoclast-like giant cells, Breast cancer, Lobular carcinoma

## Abstract

**Background:**

Breast carcinoma with osteoclast-like giant cells (OGCs) is infrequent, being most reported cased described as ductal invasive carcinomas. Invasive pleomorphic lobular carcinoma (PLC) is a distinct morphological variant of invasive lobular carcinoma characterized by higher nuclear atypia and pleomorphism than the classical type. In the best of our knowledge, a PLC with OGCs has not been previously reported.

**Case presentation:**

We report the case of a 72-year-old woman presenting with a pleomorphic tumor of the left breast with a dense infiltration by OGCs and T lymphocytes with a 10:1 predominance of CD8+ over CD4+ cells. The diagnosis of a lymphoid or mesenchymal neoplasia was excluded after demonstrating keratin expression by the neoplastic cells. The absence of E-cadherin expression and the morphological features were consistent with the diagnosis PLC with OGCs. In addition, we demonstrated the deleterious mutation C.del866C in CDH1gene, but no mutations in any of the other 33 genes analyzed by next generation sequencing.

**Conclusions:**

Breast carcinoma with stromal osteoclast-like giant cells is a very rare tumor, for that reason, the use of the cytologic features and growth patterns in combination with immunohistochemically studies is mandatory for a correct diagnosis of lobular carcinoma. In addition, further studies are necessary to clarify the influence of OGCs in the prognosis of these patients.

**Electronic supplementary material:**

The online version of this article (10.1186/s13000-018-0744-6) contains supplementary material, which is available to authorized users.

## Background

Breast carcinoma with stromal osteoclast-like giant cells (OGCs) was described by Agnatis and Rosen in 1979 [1], and accounts for 0.5–1.2% of all carcinomas of the breast [1–3]. Although the origin of the OGCs is controversial, immunohistochemical studies suggest a benign histiocytic origin as a response to cytokines produced by tumor cells in the process of angiogenic stimulation [1, 2, 4–9].

The majority of infiltrating breast carcinomas with OGCs described in the literature are invasive ductal carcinomas [1–3, 10]. Despite being reported in other subtypes, to date, there have been few reported cases of invasive lobular carcinomas with OGCs [1, 6–9].

The aim of this study is to report the first pure pleomorphic lobular carcinoma of the breast with osteoclast-like giant cells in a 72-year-old woman. The diagnosis of lobular carcinoma was confirmed by immunohistochemistry (loss of expression of E-cadherin), and by targeted next generation sequencing, which identified a *CDH1* mutation. Immunohistochemical analysis demonstrated a predominant histiocytic and T lymphocyte inflammatory response and no expression of PDL-1 in tumor or inflammatory cells. In addition, we present a literature review of the association of lobular carcinoma and OGCs.

## Case presentation

A 72-year-old woman was referred to our hospital with one-month history of a palpable mass with burning sensation in her left breast. Mammography revealed a nodular increased density of the upper inner quadrant of the left breast considered to be suspicious of malignancy, Breast Imaging Reporting and Data System category 5 (BI-RADS-5).

Ultrasound revealed a hypoechoic mass with irregular and poorly defined margins measuring 23 mm × 14 mm. The ipsilateral axillary lymph nodes were normal. After a diagnosis of malignancy on core needle biopsy, the patient underwent simple mastectomy of the left breast and sentinel lymph node biopsy.

**Fig. 1:**
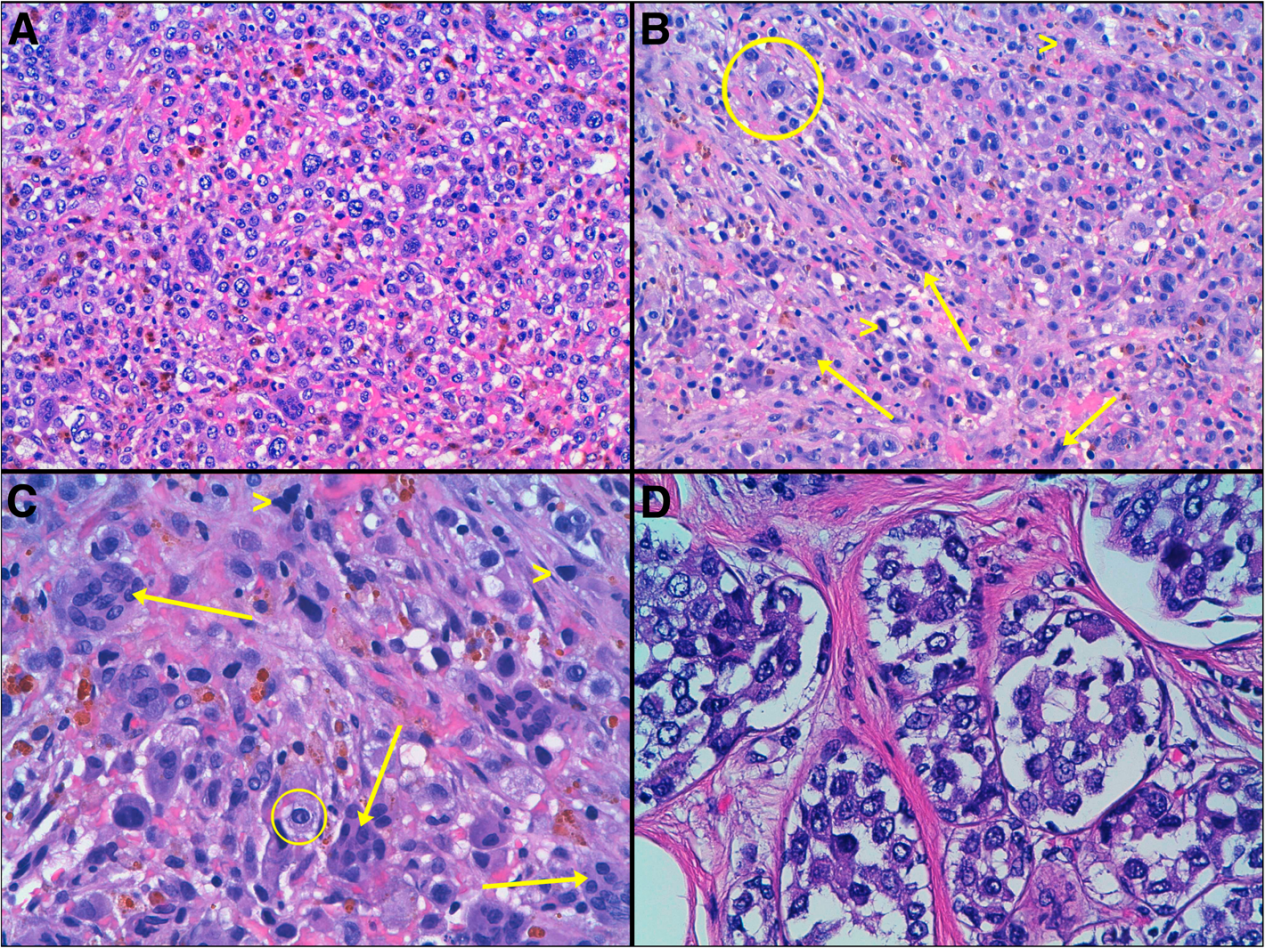
Histological findings (H-E). **a** 20× magnification, Heterogeneous proliferation of cells in a highly vascular stroma with hemorrhagic areas, hemosiderin-laden macrophages and chronic inflammatory infiltration. **b** 20× magnification and **c** 40× magnification. Numerous osteoclast-like giant cells that vary in shape and size with an eosinophilic cytoplasm (arrows).Marked nuclear atypia: Vesicular appereance, hyperchromatic (head of arrow) or with prominent nucleolus (circle). **d** 40× magnification, Focus of carcinoma in situ with the same nuclear atypia

On gross examination, two neighboring foci were found measuring together 28 mm × 17 mm. There were ill-defined whitish lesions with soft red-brown areas inside. No nipple and periareolar lesion were seen. Histologically, both tumor foci were identical, and similar features were observed in the 6 sections examined. The tumor showed high cellularity arranged in sheet of discohesive cells with large cytoplasm and marked nuclear atypia. The tumour cells showed 15 mitosis per high power microscopic field. The lesion included numerous osteoclast-like giant cells containing many small uniform nuclei and hemosiderin-laden macrophages. The stroma was loose, highly vascular with hemorrhagic areas and foci of chronic inflammatory infiltration. Some carcinomas in situ foci were also identified at the periphery of the infiltrating tumour (Fig. 1).

One of the three sentinel lymph nodes analyzed using routine intraoperative One-Step Nucleic Acid Amplification (OSNA) assay showed metastasis (17,000 copies/uL). Subsequently, ipsilateral axillary dissection was performed and no additional metastases were found in 14 additional lymph nodes resected.

**Fig. 2:**
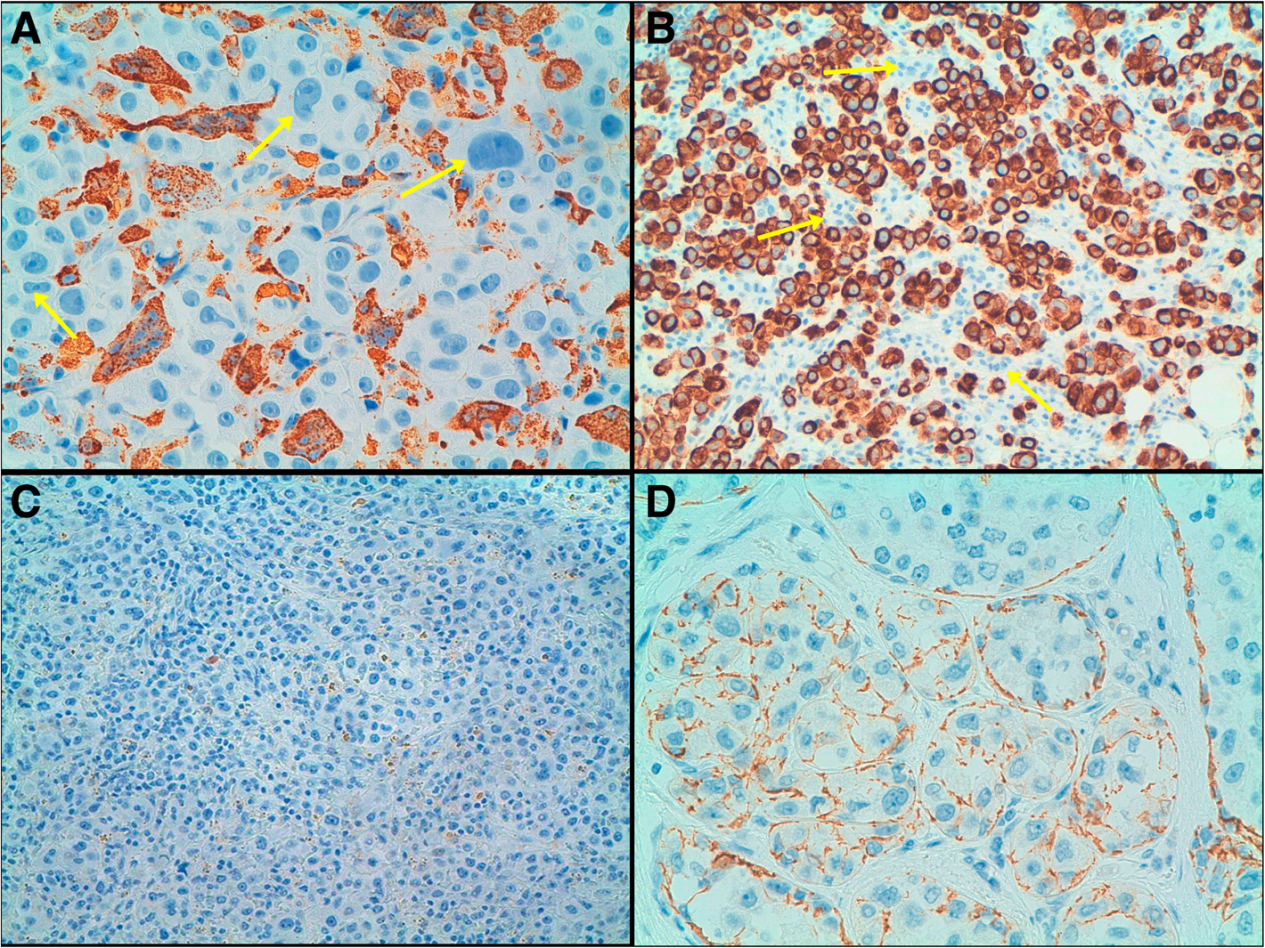
Inmunohistochemistry. **a** Osteoclast-like giant cells were positive for CD68. In contrast, neoplastic cells were negative for CD68 (arrows). **b** Neoplastic cells were positive for cytokeratin (CK) AE1/AE. In contrast, Osteoclast-like giant cells were negative for CK AE1/AE3 (arrows). Both (**c**) the invasive component and (**d**) the in situ component showed complete absence of expression for E-cadherin

The immunohistochemical study (see Additional file 1: Table S1) demonstrated the epithelial nature of the neoplasia, since the tumour cells expressed both cytokeratins AE1/AE3 and CK19 that were positive. Due to the discohesive nature of the cells, immunostaining for E-cadherin was performed and demonstrated complete absence of expression in both, the in situ and the invasive components. On the contrary, giant cells were negative for cytokeratin expression but were strong positive for the histiocytic marker CD68. With these features, the diagnosis was of invasive pleomorphic lobular carcinoma (histological grade 3) with OGCs (Fig. 2).

Biomarker analysis demonstrated that the carcinoma was estrogen receptor (ER) positive (strong positivity in 90% of tumor cells); progesterone receptor (PR) negative (complete absence of expression); and demonstrated lack of overexpression of human epidermal growth factor receptor type 2 (HERCEPTEST 1+). The Ki67 cellular proliferation index was 18%. The analysis of immune related markers demonstrated that, after counting at least 10HPF, the tumors has a mean of 34 CD3+ lymphocytes per 1HPF, 22 CD8+ lymphocytes per 1HPF, 2 CD4+ lymphocytes per 1HPF, and 1 CD20+ lymphocyte per 1HPF. Only occasional tumor cells (less than 1%) were PDL-1 + .

**Fig. 3:**
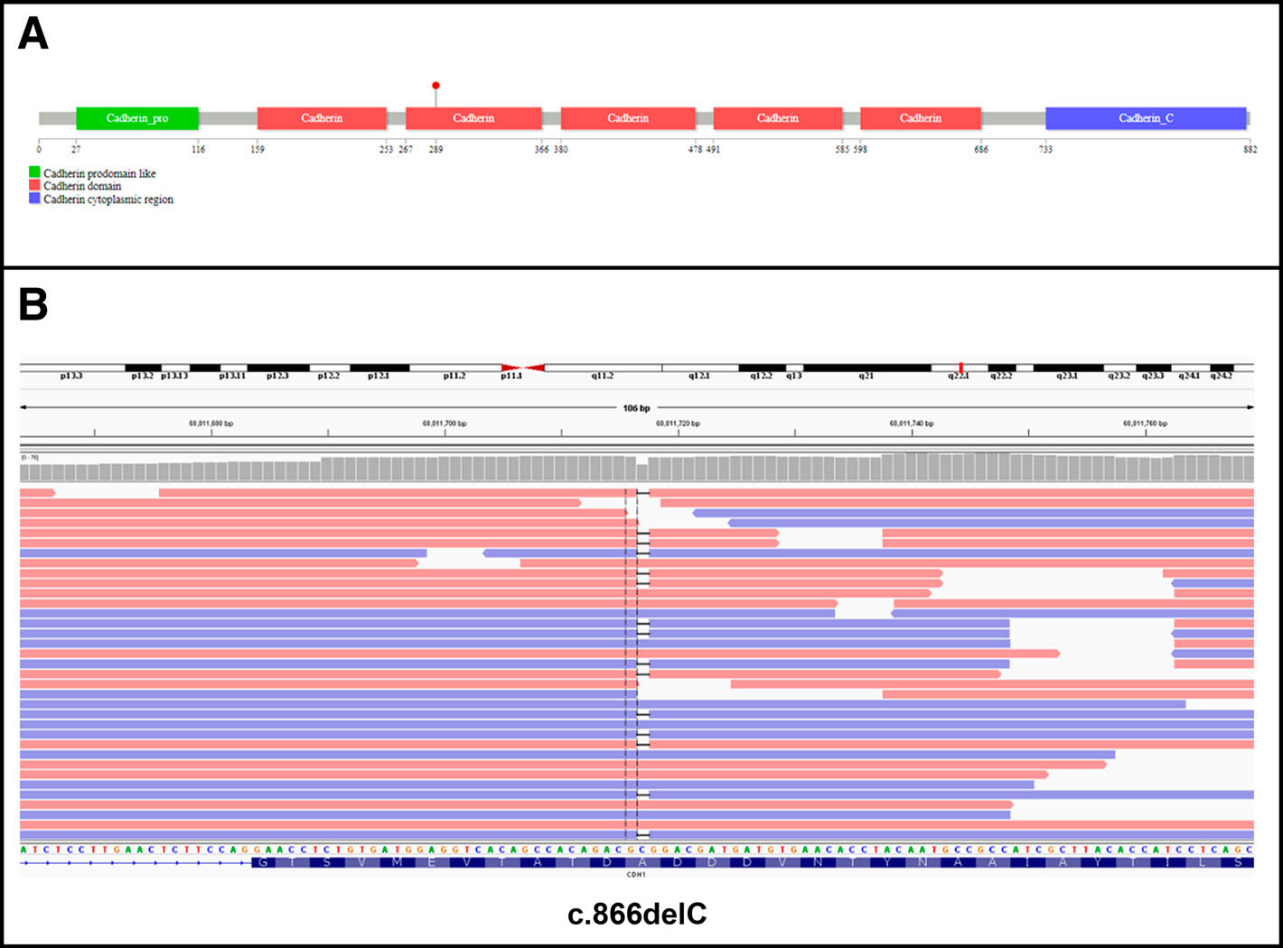
Deleterious mutation in CDH1 found in the case studied. **a** Lollipop representation of deleterious mutation c.866delC in CDH1. E-cadherin motifs are represented in different colours. **b** IGV-browser view of mutation c.del866C through massive sequencing data from the case studied. Mutation is represented in both DNA strands (5′- > 3′, red, and 3′- > 5′, blue) in a high frequency of reads, supporting a homozygous status (through LOH) within tumour cells

The tumor was subjected to molecular analysis by targeted next generation sequencing. DNA was extracted from a punch focused on the area with greater tumour cell density by using QIAamp® DNA FFPE Tissue Kit (QIAGEN). Quantification (447 ng/μl) and qualification (DIN = 4.6) of the DNA was performed using a Tape Station 2200 (Agilent) and the Genomic DNA kit. We used an in-house panel based on hybrid capture for sequence enrichment including the 34 genes frequently mutated in breast cancer (*AKT1, ARID1A, ARID1B, BRCA1, BRCA2, CASP8, CCND1, CDH1, ERBB2, ESR1, FGFR1, GATA3, GRB7, GSDMB, MAP2K4, KRAS, MAP3K1, MLL3, MYC, NCOR1, NF1, PGAP, PIK3CA3, PNMT, PTEN, RB1, SF3B1, STARD3, TBX3, TCAP, TP53, VGLL1, ZNF217, ZNF703*) and regions in chromosome 8 (targeting amplification of *FGFR1* and *MYC*), chromosome 11 (targeting amplification of *CCND1*), chromosome 17 (targeting amplification of *ERBB2*) and chromosome 20 (targeting amplification of *ZNF217*). With this technique, we identified a deleterious mutation (C.del866C) in *CDH1* (the gene coding for E-cadherin protein) (Fig. 3).

The patient was enrolled in a trial that studies giving tamoxifen with or without combination chemotherapy in postmenopausal women who have undergone surgery for breast cancer. The patient was randomized on the arm for receiving only hormone treatment. The patient remains well without evidence of recurrence or metastases two years after surgery.

## Discussion

The differential diagnosis in present case included both lymphoid and mesenchymal neoplasias, due to the diffuse growth pattern, the abundance of inflammatory cells and the presence of OGCs. However, the expression of cytokeratins in pleomorphic neoplastic cells allowed the diagnosis of invasive breast carcinoma. The histological type of present tumor was only suspected after the observation of the in situ component, which was concordant with an in situ lobular carcinoma. The absence of E-cadherin expression in both the in situ and invasive components, the cytological features of the tumors and the presence of CD68-positive OGCs were consistent with the final diagnosis of pheomorphic lobular Carcinoma with OGCs.

Breast carcinoma with osteoclast-like giant cells is a rare entity that accounts for less than 2% of all carcinomas of the breast [1–3]. After its first description by Agnatis and Rosen in 1979 [1], approximately 200 cases have been published. Among the histological types of breast carcinoma with OGCs that have been reported, invasive ductal carcinoma is the most frequent [1–3, 10]. In the best of our knowledge, only 7 previous invasive lobular carcinoma (ILC) with OGCs have been described in literature, but none of these had confirmation of the histological type by E-cadherin immunohistochemistry and/or mutational analysis of *CDH1 gene* nor were of pleomorphic subtype. (Table 1) [1, 6–9]. Regarding the histological subtypes of previous ILC with OGCs, 5 cases were described as classical ILC (one of them bilateral) and two as alveolar ILC. Two reported cases were mixed invasive ductal and invasive lobular carcinomas with OGCs. Since there is no description of E-cadherin staining in these reports, the possibility of ductal carcinoma with areas of “lobular” growth cannot be excluded. In this sense, it has been reported that more than two third of mixed ductal-lobular carcinomas do not have *CDH1* mutations and their mutational and transcriptional profiles are suggestive of invasive ductal carcinoma [11].

**Table 1:** Summary 7: published cases of invasive lobular carcinoma of the breast with Osteoclast-like giant cells

Reference	Yr of publication	Age (yr)	Site	Size (cm)	Type of carcinoma
Agnatis 1	1979	50	NA	0.5	ILC, IDC
		43	Left (UOQ)	3.6	ILC
		48	Left (UOQ)	5.0	ILC, IDC
Pettinato 7	1989	40	Right (UOQ)	2.0 × 2.0	ILC alveolar variant
Reale 8	1993	63	Left (LOQ)	3.5	ILC alveolar variant
Takahashi 9	1998	48	Right (UOQ)	1.5 × 1.5	ILC
Iacocca 10	2001	46	Left (UIQ)	11 × 5.9	ILC
			Right	–	–

*NA* not available, *UOQ* upper outer quadrant, *LOQ* lower outer quadrant, *UIQ* upper inner quadrant, *ILC* invasive lobular carcinoma, *IDC* invasive ductal carcinoma

Pleomorphic Lobular Carcinoma (PLC) was first described in 1987 by Page and represents less than 1% of all invasive breast carcinomas [12, 13]. However, the diagnostic criteria of PLC are not fully defined. The World Health Organization (WHO) [14] describes it as a variant of the ILC that retains the distinctive growth pattern of classic lobular carcinoma, but showing a greater degree of cellular atypia and pleomorphism and a higher mitotic rate than classic ILC. As for classic ILC, loss of expression of E-cadherin, together with *CDH1* LOH, is a nearly constant finding of PLC [15, 16].

In addition to the absence of E-Cadherin expression, we demonstrated the *CDH1* mutation 866delC by targeted next generation sequencing. No other alterations were observed in any of the remaining 33 genes analyzed for mutations or in the 5 regions analyzed for gene amplification. Recently, three large studies [11, 17, 18] have reported the molecular alterations of lobular carcinomas. Although with some differences among studies, some of the most common alterations detected were mutations in CDH1 (42.8% to 65%), PIK3CA (34.8% to 48%), TBX3 (9% to 13.3%), FOXA1 7% to (9%), GATA3 (5% to 7.1%), MAP3K1 (5.1% to 6%), and AKT1 (2.5% to 5.1%). Compared with classical ILC, a higher frequency of HER2 mutations have been identified in PLC (20.8%) [19].

A recent genome-wide gene expression study [18] suggested the existence of two main subtypes of ILC: an immune related subtype (IR), characterized by lymphocytic infiltration and up-regulation of “checkpoint proteins”, and a hormone related subtype, characterized by active ER/PR signaling and Epithelial to Mesenchymal Transition (EMT). The IR subtype showed high expression of numerous cytokine/chemokine signaling pathway components found in lymphoid cells, and over-expression of CD8 and CD4. Although we did not perform gene expression analysis, probably present case represents an example of IR-ILC, since our immnuhistochemical study demonstrated, together with a high number of OGCs, a heavy infiltration by T lymphocytes with 10:1 predominance of CD8+ over CD4+ cells. Although as a group the IR tumours also had high expression of the negative regulators of immune response PDCD1 (PD-1), CD274 (PD-L1) and CTLA4 (CTLA-4), immunohistochemical expression of these markers was only observed in some cases. According to these observations, our case did not show PDL-1 expression in tumor or inflammatory cells.

The prognostic significance of the presence of OGCs in breast carcinomas, remains controversial since some authors suggested a less favorable prognosis for invasive breast cancer with OGCs [1, 2], whereas others reported a similar or better prognosis than the infiltrative carcinomas without OGCs [10, 18]. Given this discrepancy, it is probably that the prognosis is more related to the type of cancer associated than to the presence or not of the OGCs. In this sense, it has been described that PLC has a worse prognosis than classical ILC [13, 20–22].

## Conclusion

- Breast carcinoma with stromal osteoclast-like giant cells is a very rare tumor. Among the few reported cases, the association of OGCs with invasive lobular carcinoma is even rarer.
- It was of importance the use of the cytologic features and growth patterns in combination with inmunohistochemical studies, in this case, loss of E-cadherin expression, for a correct diagnosis of lobular carcinoma.
- The mechanism of formation of osteoclast-like giant cells is still unknown. However, most of the cases described support a non-epithelial, stromal histiocytic origin.
- The influence of OGCs in the prognosis of patients is still controversial. Nonetheless some studies suggest that the prognosis may be more related to the type of cancer associated rather than the presence or not of the OGGs.
- Further studies are needed to gain more comprehension of this rare tumor and determine future treatment strategies.

## Additional file

Additional file 1: **Table S1.** Antibodies used for the immunohistochemical study of case reported. (DOCX 16 kb)

## Abbreviations

ILC: Invasive lobular carcinoma
OGCs: Osteoclast-like giant cells
PLC: Pleomorphic lobular carcinoma
