# Supplementary material for: Pleomorphic lobular carcinoma of the breast with osteoclast-like giant cells: a case report and review of the literature
Source: Diagn Pathol. 2018 Aug 28;13:62. doi: 10.1186/s13000-018-0744-6 (PMC6114493; doi:10.1186/s13000-018-0744-6)
Supplement: Supplementary file 1 — Table S1. Antibodies used for the immunohistochemical study of case reported. (DOCX 16 kb) [file 13000_2018_744_MOESM1_ESM.docx]

**TABLE S1: Antibodies used for the immunohistochemical study of case reported.**

| **Target** | **Trade** | **Clone** | **Dilution** | **#Cat.** |
| --- | --- | --- | --- | --- |
| E-cadherina | Dako , Glostrup,Denmark | IR059 | ready to use | IR059 |
| Cytokeratin AE1/AE3 | Dako , Glostrup,Denmark | AE1/AE3 | ready to use | GA053 |
| Cytokeratin 19 | Dako , Glostrup,Denmark | RCK108 | ready to use | IR615 |
| CD68 | Dako , Glostrup,Denmark | PGM1 | ready to use | GA613 |
| CD3 | Dako , Glostrup,Denmark | Polyclonal | ready to use | GA503 |
| CD8 | Dako , Glostrup,Denmark | C8/114B | ready to use | GA623 |
| CD4 | Dako , Glostrup,Denmark | 4B12 | ready to use | IR649 |
| CD20 | Dako , Glostrup,Denmark | L26 | ready to use | GA604 |
| PDL-1 | Dako , Glostrup,Denmark | 22C3 | ready to use | SK006 |
| Ki67 | Dako , Glostrup,Denmark | MIB-1 | ready to use | M7240 |
| ER | Dako , Glostrup,Denmark | EP1 | ready to use | IR059 |
| PR | Dako , Glostrup,Denmark | PgR 636 | ready to use | IR068 |
| Her 2 | Dako , Glostrup,Denmark | Polyclonal | ready to use | SK001 |
